# Fracture Load of Metal, Zirconia and Polyetheretherketone Posterior CAD-CAM Milled Fixed Partial Denture Frameworks

**DOI:** 10.3390/ma14040959

**Published:** 2021-02-18

**Authors:** Verónica Rodríguez, Celia Tobar, Carlos López-Suárez, Jesús Peláez, María J. Suárez

**Affiliations:** Department of Conservative Dentristy and Bucofacial Prosthesis, Faculty of Odontology, University Complutense of Madrid, 28040 Madrid, Spain; veranicr@ucm.es (V.R.); cetobar@ucm.es (C.T.); carlop04@ucm.es (C.L.-S.); mjsuarez@ucm.es (M.J.S.)

**Keywords:** zirconia, PEEK, CAD-CAM, fracture load, fixed partial dentures

## Abstract

The aim of this study was to investigate the load to fracture and fracture pattern of prosthetic frameworks for tooth-supported fixed partial dentures (FPDs) fabricated with different subtractive computer-aided design and computer-aided manufacturing (CAD-CAM) materials. Materials and Methods: Thirty standardized specimens with two abutments were fabricated to receive three-unit posterior FDP frameworks with an intermediate pontic. Specimens were randomly divided into three groups (n = 10 each) according to the material: group 1 (MM)—milled metal; group 2 (L)—zirconia; and group 3 (P)—Polyetheretherketone (PEEK). The specimens were thermo-cycled and subjected to a three-point bending test until fracture using a universal testing machine (cross-head speed: 1 mm/min). Axial compressive loads were applied at the central fossa of the pontics. Data analysis was made using one-way analysis of variance, Tamhane post hoc test, and Weibull statistics (α = 0.05). Results: Significant differences were observed among the groups for the fracture load (*p* < 0.0001). MM frameworks showed the highest fracture load values. The PEEK group registered higher fracture load values than zirconia samples. The Weibull statistics corroborated these results. The fracture pattern was different among the groups. Conclusions: Milled metal provided the highest fracture load values, followed by PEEK, and zirconia. However, all tested groups demonstrated clinically acceptable fracture load values higher than 1000 N. PEEK might be considered a promising alternative for posterior FPDs.

## 1. Introduction

In the last few decades, developments in materials and technologies had supposed an advance in the fabrication of fixed dental prostheses (FDPs) [1,2]. Computer-aided design and computer-aided manufacturing (CAD-CAM) technology allows for superior results to be obtained, with more time efficiency, an improvement in cost/effectiveness, and higher-precision prostheses compared to conventional manufacturing techniques [3]. CAD-CAM techniques involve additive, or layer-by-layer, and subtractive manufacturing processes [2,3]. Subtractive technology is based on processes in which machines with motorized tools, such as saws, lathes, milling machines, and drills, are used to mechanically cut solid blocks and achieve the desired geometry as controlled by a software program. The main advantage of subtractive technology is the ability to create complex geometries with no imperfections [4]. However, its main drawbacks are the expensive machinery, the working time, the wear suffered by the equipment, and the large amount of residual and non-recoverable material. Most dental materials are available for machining, which is an additional advantage compared to other CAD-CAM technologies [5].

Despite the advances of CAD-CAM technology, the success of the restorations is determined by three main factors: marginal fit, fracture resistance, and esthetics [6]. Moreover, mechanical properties and load to fracture are important factors to determinate the use of a restorative material that should support chewing forces and protect the tooth structure. In addition, in the oral cavity, temperature variations occur constantly that can produce important alterations in the material’s strength. Therefore, artificial aging is recommended during in vitro studies to simulate the oral environment conditions [7,8,9].

To date, cobalt–chromium (Co-Cr) alloys are widely used for the fabrication of prosthetic frameworks [2,3]. When processed by CAD-CAM technology it allows for obtaining more homogeneous and refined microstructures, assuming an additional improvement in their mechanical properties [10]. However, the main material’s goal is to achieve an improvement in esthetic, without decreasing resistance. High-strength ceramics such as zirconia fabricated with CAD-CAM technologies were developed to eliminate metal frameworks [11]. Zirconia offers high toughness, fracture strength, and reliability, acquiring a major role in the manufacture of frameworks for crowns and fixed partial dentures (FPDs), even in the posterior regions [12]. However, it is a very opaque material that must be covered with feldspathic ceramic to improve the esthetic results [11,13]. However, the interface between the two ceramics is one of the weakest aspects, and delamination and chipping of the veneering ceramic is the main failure mode of bilayer zirconia restorations [11,12,13,14]. A recent study evaluated the zirconia–veneer interface and reported new insights that explain the high chipping rates observed [15]. Successive generations of zirconia have been developed to solve the chipping problem of the first-generation zirconia and to improve its translucency [11,14]. 

Currently, new polymeric materials have been developed to fabricate dental frameworks. These materials have different matrix composition and several percentages of ceramic or resin [16]. Polymeric materials are available in monolithic blocks for CAD-CAM technology, presenting better properties than manually processed polymeric materials [16]. 

Polyetheretherketone (PEEK) is the most used polymer in the dental area, and has a low modulus of elasticity (3–4 MPa), similar to human cortical bone. In addition, it is characterized by its biocompatibility, dimensional stability, and higher fracture strength than other plastic materials used in dentistry, even when subjected to temperature variations [17]. However, it is not esthetic enough due to its greyish-brown color, and veneering is essential [18]. Nevertheless, veneering with light-curing composites makes the chipping clinically repairable and prevents the wear of opposing teeth [19,20]. PEEK is a relatively new material that is becoming widespread in clinical practice, although few studies are available on CAD-CAM FPDs [21].

Therefore, the aim of this study was to evaluate and compare the fracture load and fracture patterns of metal, zirconia, and PEEK 3-unit posterior CAD-CAM milled FPD frameworks. The null hypotheses tested were that no differences would be found in load to fracture among the materials, and that the fracture patterns would not differ among the materials.

## 2. Materials and Methods

### 2.1. Preparation of Specimens

Thirty standardized specimens with two abutments and a base were machined in stainless steel 316L Alloy (UNS S3 1603) rods in the Mechanical Workshop of the Physical Science Faculty (University Complutense of Madrid, Madrid, Spain). The abutments’ configuration was 5 mm in height, 6° angle of convergence, 1 mm width chamfer, and rounded angles to simulate clinical conditions. The bases were designed as follows: 30 mm in length, 4.5 mm in height, 17 mm in width, and with 2 centered perforations separated by 7 mm [1,6,8,13,22]. The features of the specimens were introduced in the design program (AutoCAD 2011; Autodesk, San Rafael, CA, USA). The specimens were manufactured using the EMCO Turn 343 numerical control lathe (EMCO Group; Hallein, Austria) governed by a software (SINUMERIK; Siemens AG; Munich, Germany) [23]. The dies were randomly screwed onto the metallic base to receive posterior 3-unit FPDs with an intermediate pontic, so that one of them simulated a first mandibular premolar and the other a first mandibular molar.

Three types of commercially available CAD-CAM milled materials were used. The specimens were randomly assigned to three groups (n = 10 each according to the results of power analysis) categorized according to the materials used to fabricate the FPD frameworks: MM—milled Co-Cr (Starbond CoS Disc basic; Scheftner, Mainz, Germany); L—zirconia (Lava Zirconia; 3M-ESPE, Seefeld, Germany) and P—milled PEEK (Bio-P; DEGOS Dental, Regenstauf, Germany). 

To fabricate the MM frameworks the specimens were scanned and digitized (D750 scanner; 3Shape, Copenhagen, Denmark) and the data were entered into specific design software (CAD Molder Builder; 3Shape) [24]. The frameworks were designed with a thickness of 0.5 mm, a rounded connector of 9 mm^2^ (3 mm × 3 mm), and an internal space of 50 µm for the luting agent. These parameters were programmed with the aforementioned software. The frameworks were milled from sintered Co-Cr discs in the milling unit (Ultrasonic 10 linear; SAUER-DMG Mori, Stipshausen, Germany) according to the manufacturer’s instructions. The alloy composition was as follows: Co—59%; Cr—25%; W—9.5%; Mo—3.5%; Si—1%; C, Fe, Mn, and N—≤1.5%. After milling, the specimens were cleaned with water steam and sandblasted with 150 µm alumina particles for 10 s at a pressure of 2 bar to remove the surface contaminants.

Zirconia frameworks (first-generation zirconia) were digitized (Lava Scan ST; 3M ESPE, Seefeld, Germany) and designed (Lava Design Software; 3M ESPE). The same parameters as in the metal group were programmed into the software. The design was enlarged by 20% to offset post-sintering shrinkage [8,13,22,23]. Manufacturing was performed from pre-sintered zirconia blocks using the milling unit (Lava Form; 3M ESPE), and sintered after milling in a specific furnace (Lava Therm; 3M ESPE) at 1500 °C for 4 h [13,22,23].

PEEK frameworks were scanned (Lava Scan ST; 3M ESPE) and designed with specific CAD software (DWOS Lava Edition; Dental Wings, Montreal, QC, Canada). The following parameters were programmed: internal space of 50 µm for the cement, wall thickness of the copings of 0.7 mm, and rounded connectors of 16 mm^2^ (4 mm × 4 mm), following the manufacturer’s instructions [25]. The frameworks were milled from PEEK discs in the milling unit (Yenadent D43; Yenadent, Istanbul, Turkey).

A specialist technician calibrated each milling unit before milling. After milling, the thickness of each framework was verified by taking measures at different locations with a digital micrometer (Mitutoyo Co; Tokyo, Japan) with an accuracy of 0.01 mm [13,22].

All frameworks were luted with glass ionomer cement (Ketac-Cem EasyMix; 3M-ESPE) in standard fashion to their corresponding master dies by the same operator at room temperature (18 to 24 °C) and 50 ± 10% relative humidity [1,8,13,22]. A constant seating load of 10 N was applied for 10 minutes with a torque wrench (Ziacom, Madrid, Spain) fitted to a customized device (Mechanical Workshop of Physical Science, University Complutense of Madrid) [13,22].

### 2.2. Mechanical Test

After 48 h of water storage, each group was subjected to thermal cycling in a climatic chamber (CCK0/81; Dycometal, Viladecans, Spain) controlled with Eurotherm iTools software (Eurotherm, Worthing, UK). The thermo-cycling was performed in Fusayama-Meyer artificial saliva (LCTech, Obertaufkirchen, Germany). Each specimen underwent 6000 thermal cycles at 5 °C and at 55 °C. All frameworks were then further subjected to a three-point bending test until fracture (National Center for Metallurgical Research-CENIM; CSIC, Madrid, Spain) using a universal testing machine at a crosshead speed of 1 mm/min (ME 405/10; SERVOSIS, Pinto, Spain) [1,8]. Axial compressive loads were applied at the central fossa of the pontics until the fracture initiation of the restorations, defined by a sharp fall in the loading curve, together with the visible fracture of the framework [1,8,13,22]. Data on the load to fracture were automatically recorded in Newtons (N) using a software program (PCD2K; SERVOSIS) that allowed force (N)–displacement (mm) curves to be created [1,8,13,22].

### 2.3. Statistical Analysis

Means and standard deviations (SD) were calculated for each group. The Shapiro–Wilk test confirmed that the data were normally distributed. One-way analysis of variance and a Tamhane test were performed for comparisons of the load to fracture among the groups. In addition, the Weibull characteristic fracture load (0) and the Weibull modulus (m) were also analyzed [8,13,25]. Statistical analysis was performed with statistical software (IBM SPSS Statistics, v22.0; IBM Corp, Armonk, NY, USA). Statistical significance was set at α = 0.05.

## 3. Results

**Figure 1:**
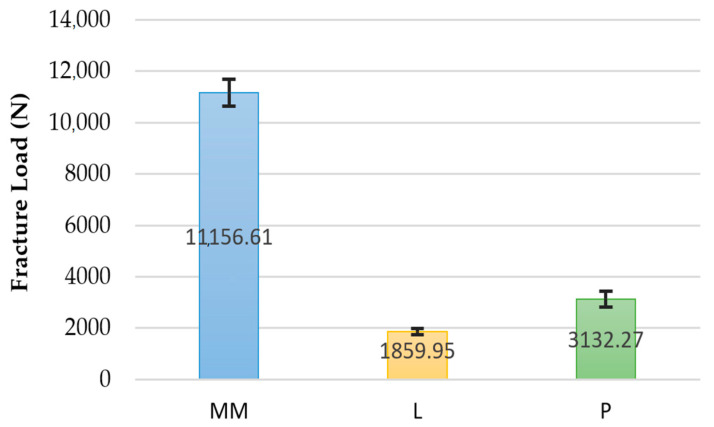
Graph of fracture load values among the groups (MM: milled metal; L: zirconia; P: PEEK).

**Figure 2:**
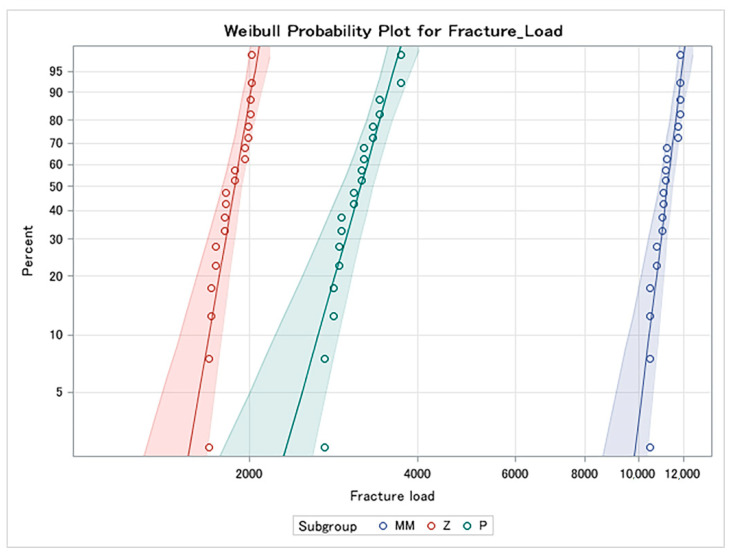
Weibull probability plot of the fracture load for Milled Metal, Zirconia, and PEEK groups.

Table 1 and Figure 1 display the mean load to fracture values for the experimental groups. All materials tested recorded load to fracture values higher than 1000 N. ANOVA revealed significant differences among the groups (*p* < 0.0001; f = 1941.86). Tamhane’s post hoc test indicated that the load to fracture of the MM group was significantly higher than the other groups (*p* < 0.0001) (Table 2). These data were corroborated by the Weibull distribution parameters (Figure 2). No significant differences were found in the shape parameter (m), and this means that the behavior of the data is more predictable and that the sample is not very variable. However, significant differences were observed in the scale parameter (σ0) for all tested groups. The MM group presented the highest σ0 values (11,398.64 N). This means that 63.2% of the samples will fracture at 11,398.64 N, achieving the highest probability of survival. The zirconia group presented the lowest values (1917.04 N).

**Figure 3:**
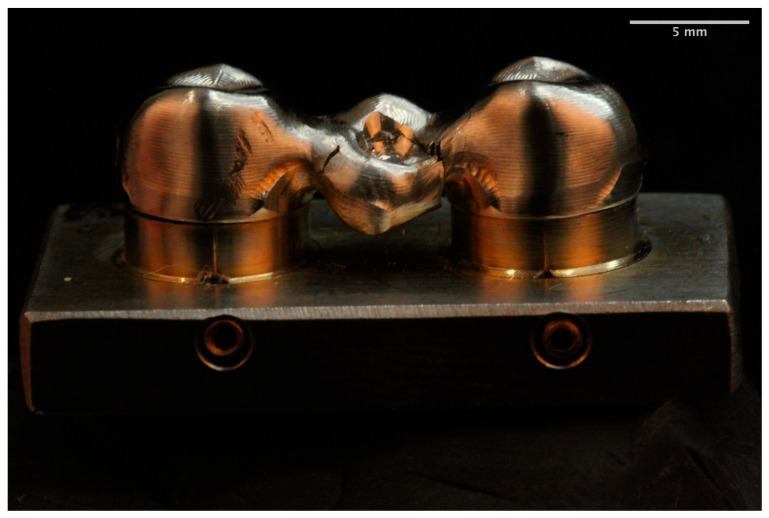
Milled metal framework fracture.

**Figure 4:**
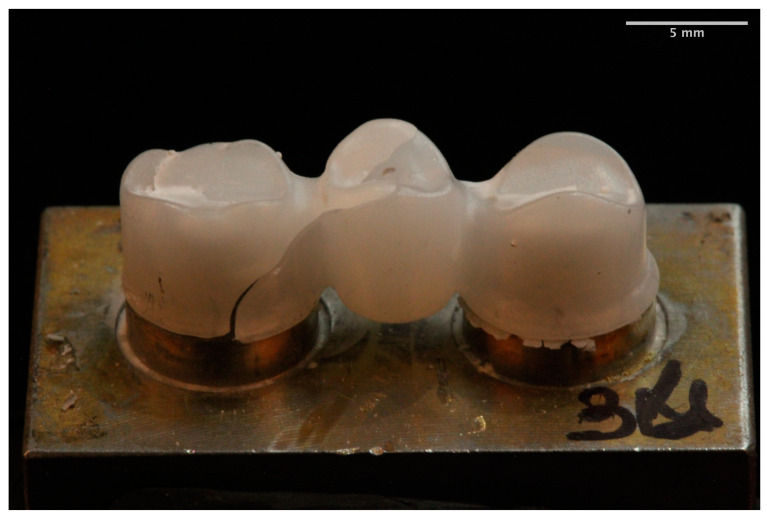
Zirconia framework fracture.

**Figure 5:**
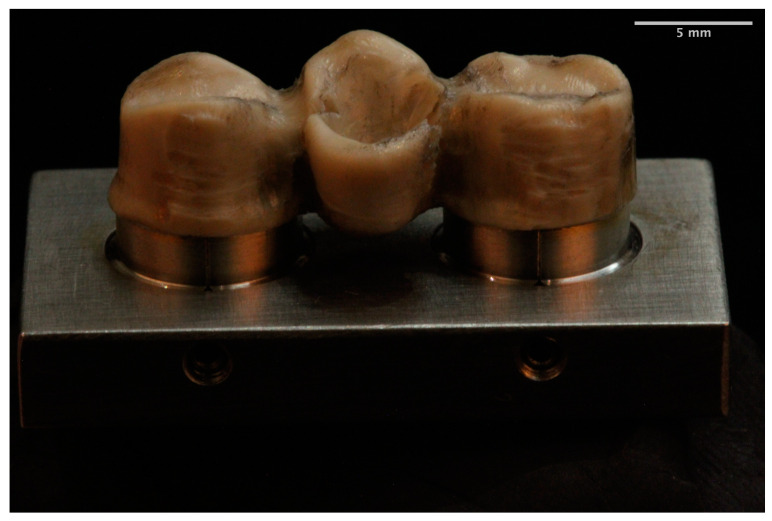
PEEK framework fracture.

The fracture pattern was different for each of the materials. The MM group showed a ductile failure starting at the gingival surface of the connectors in all frameworks [22] (Figure 3). In the zirconia group the fracture mainly occurred (80%) at the loading point through one or both connectors. The fracture started at the cervical area of the connectors and spread diagonally toward the occlusal surface of the pontic [1,22] (Figure 4). In the remaining 20% of the frameworks the fracture arose at the axial surface of one of the retainers. The PEEK group showed a ductile fracture. The cracks began in the upper zone of the connectors in all frameworks. Plastic deformation was observed without total fracture (Figure 5).

## 4. Discussion

This in vitro study evaluated and compared the load to fracture and the fracture pattern of three different materials manufactured by the CAD-CAM milled technique to fabricate posterior FPD frameworks. The results obtained in the study support the rejection of the null hypotheses, because significant differences were observed among the materials analyzed.

The mechanical properties of the materials are important criteria for selecting the restorative materials in fixed prostheses, even more so when they involve the posterior regions, since their resistance will be directly related to long-term success. The intensity of the masticatory forces greatly varies depending on several factors, including the presence of parafunctions. Parafunctional forces can reach 1000 N and this limit should be taken as a reference to ensure the resistance of the restorative materials [22,26]. In the study, all of the materials tested showed load to fracture values higher than 1000 N, thus being able to withstand clinical chewing loads.

The authors are unaware of previous studies comparing the fracture resistance of metallic, ceramic, and polymer FPD frameworks. In the present study, the MM group showed the highest load to fracture values compared to zirconia and PEEK groups, exceeding 10,000 N. The results obtained were higher than those of previous studies on Co-Cr frameworks [1,8,22]. The reasons for such differences could be the different manufacturing process [10,27], and the connector area employed (9 mm^2^).

Zirconia is the most suitable ceramic for manufacturing FPDs in the posterior regions due to its high strength [11,14]. However, in the study the zirconia group obtained the lowest load to fracture values (1859 N). The results were consistent with those of previous studies that reported similar values on FPDs with the same zirconia system [1,8,12,22,28,29]. Nevertheless, different values were reported for crowns. Choi et al. [30] found values above 4000 N, Yildiz et al. [31] obtained values slightly higher than 2000 N, and Silva et al. [32] reported values of 1134 N. Most of these studies compared Lava Zirconia with other zirconia systems, without agreeing on the zirconia system that had higher fracture resistance. Several studies found differences between different zirconia systems [13,26,29]. Conversely, other studies did not demonstrate the difference between them [31,33,34,35,36,37,38]. 

The PEEK group obtained fracture load values of 3132 N. Few studies have evaluated the fracture resistance of milled PEEK, reporting lower values than in the present study. Addullah et al. [39] reported values of 802 N in milled PEEK crowns without aging. Stawarczyck et al. [40] reported values of 1383 N on uncemented three-unit milled PEEK FPDs. In a posterior study, Stawarczyck et al. [25] reported higher fracture loads (2354 N) for milled compared to pressed PEEK. Despite the few investigations, it can be stated that milled PEEK presents good mechanical properties, even above zirconia, to be used in posterior FPDs [17,25,40].

Differences in the size of the connector, the design of the structure and the methodology used can justify the discrepancies among the different studies using the same materials. Furthermore, in the study, the load value at which the fracture initiated was registered.

The present study was conducted in vitro. In vitro studies allow for evaluating the mechanical properties of the materials under standardized conditions. However, the conditions of the studies should be established since they can influence the results. Some studies used resin dies because their elasticity modulus is similar to dentin [37,41], while others used metallic dies, as in the present study [1,6,8,13,22,25,38,42], which provide for the standardization of the shape and dimensions of the specimens [6,42], and also avoid possible premature destruction when testing metallic alloys compared to natural or resin teeth [43,44]. In the study, real frameworks were used instead of bar-shaped, cylinder, or disc specimens [29,45], and the thickness was also standardized to simulate clinical conditions [37,44,46,47,48]. Therefore, this standardization allowed for comparison with groups under the same conditions.

The inclusion of artificial aging is a controversial topic. Several authors used only static load in their studies, suggesting that compressive forces are adequate for evaluating the fracture resistance of crowns or FPDs [13,26,31,37,38]. However, other authors included artificial aging in their research to reproduce the conditions in the oral environment [8,22,28,33,35,47,49,50,51,52,53]. The results comparing both tests are controversial, since some authors found no influence of aging on the frameworks’ resistance [8,9,34,46,54,55], while others reported a decrease in fracture resistance after artificial aging [53]. Niem et al. [56] concluded that the mechanical properties of ceramic CAD-CAM materials and polymer-based materials were not affected by thermocycling in terms of their flexural strength and modulus of elasticity. Conversely, most hybrid composite materials showed significant degradation [21,56]. In addition, there are great differences among the studies in the test conditions, and no standardization exists regarding the number of cycles, the load applied, the temperatures, or the solution used (distilled water or saliva) [8,9,18,28,32,35,43,44,49,51,52,54,57]. In the present study, the specimens were subjected to 6000 thermal cycles simulating 5 years in mouth [58]. 

Knowing the fracture pattern is an important aspect of understanding the behavior of materials subjected to load. When there are bending forces on the structure, compression forces are produced on the side where the force is applied, and traction forces are produced on the opposite side. Cracks usually occur on the tensile side, and propagate to the compression side, causing fracture [59]. 

All metal frameworks showed the highest plastic deformation prior to fracture as evidenced by the force–displacement curves. No separation of the fragments was observed. According to previous studies [22,60,61], this was translated into ductile failure that was initiated in all frameworks at the gingival surface of the connector. Consistent with the present study, previous studies have reported that the connector area withstands the highest tensile and shear forces [13,33,38]. The minimal dimensions for this type of connector are 6.25 mm^2^ [61]. In order to compare the MM group with the other experimental groups, 9 mm^2^ connectors were made, which may explain the high load to fracture values obtained.

Conversely, the zirconia frameworks showed a brittle fracture in which the fragments perfectly fitted to each other along the fracture line [22]. The breakage mainly (80%) occurred at the connector’s level, demonstrating that this area supported the highest stresses [13,22,33,38,62]. The force–displacement curves showed that fracture presented a fast propagation without a previous deformation. The fracture was initiated in the gingival area of connector and propagated obliquely to the occlusal area of the pontic [1,12,13,38,44]. Likewise, a previous study reported that this pattern of fracture is independent of the loading point [12]. In the study, the connector area for zirconia frameworks was 9 mm^2^, as recommended by several authors [8,22,32,38,54,62]. This design is very important to reduce the tension and the fracture risk [12,62]. In addition, the greater the number of units, the larger the connector area should be.

Regarding PEEK frameworks, a different fracture pattern was observed. The fracture did not initiate at the connector. A plastic deformation of the pontic was observed until there were cracks on both sides in the upper area of the connectors. No separation of the fragments was observed in any of the specimens. This resulted in a ductile failure. This behavior was previously reported in PEEK frameworks manufactured by pressing [25] and milling techniques [40]. The size of the connectors used was 16 mm^2^, following the manufacturer’s recommendations. The same dimension was used by Stawarczyck et al. [25], although other studies used smaller connector areas [18,40]. Dal Piva et al. [63] reported a low elastic modulus (4 GPa) on PEEK crowns compared to Co-Cr alloys (220 GPa), gold alloys (91 GPa), zirconia (220 GPa), alumina (314 GPa), lithium disilicate (95 GPa), zirconia-reinforced lithium silicate (70 GPa), and feldspathic porcelain (48.7 GPa) [63]. Thanks to its low elastic modulus, PEEK allows the absorption of stresses derived from the function and absorbs the loads on the abutment teeth [19,20].

Limitations of the present study included its in vitro design, which may not reproduce the clinical environment. However, import aspects of clinical conditions were simulated. Furthermore, the study only analyzed the frameworks without veneering porcelain. In addition, each group required a different milling unit, and complete standardization was not possible. Considering this, the findings of the study suggest that CAD-CAM milling can be considered a suitable method for fabricating Co-Cr posterior FPD frameworks, and can be used as an alternative to the conventional casting process in terms of fracture load. Furthermore, milled PEEK may be considered a promising alternative for metalceramic or zirconia restorations in the posterior regions. However, there are not enough statements about complications, biofilm formation on PEEK surface, and its resistance to compression [64]. In addition, clinical studies are necessary to validate the results of the studied CAD-CAM systems.

## 5. Conclusions

Within the limitations of this in vitro study, the following conclusions were drawn:

1. All tested CAD-CAM milled materials demonstrated clinically acceptable fracture load values;
2. The type of material influenced the load to fracture;
3. Milled metal exhibited the highest load to facture values, followed by PEEK, and zirconia;
4. Milled PEEK could be an alternative to metal or ceramic restorations in posterior regions;
5. Different fracture patterns were observed for the analyzed materials.

## Figures and Tables

**Table 1:** Means, standard deviations (SD), maximum and minimum fracture load values (N) of all materials.

Group	N	Mean	SD	Min	Max
Milled Metal	10	11,156.61	530.69	10,430.14	11,860.94
Zirconia	10	1859.95	128.53	1687.68	2016.84
PEEK	10	3132.27	307.15	2730.28	3729.88

**Table 2:** Tamhane post hoc test. (MM: milled metal; Z: zirconia; P: PEEK).

	(I) Group	(J) Group	Mean Difference (I-J)	Deviation Error	Sig.	Superior Limit	Inferior Limit
Tamhane	MM	Z	9296.66	172.67	0.000	8803.28	9790.03
		P	8024.33	193.90	0.000	7501.07	8547.59
	Z	MM	−9296.66	172.67	0.000	−9790.03	−8803.28
		P	−1272.32	105.29	0.000	−1563.79	−980.85
	P	MM	−8024.33	193.90	0.000	−8547.59	−7501.07
		Z	1272.32	172.67	0.000	980.85	1563.79

## Data Availability

No new data were created or analyzed in this study. Data sharing is not applicable to this article.
